# Genetic diversity of phytoplasma strains infecting chrysanthemum varieties in India and their possible natural reservoirs

**DOI:** 10.1007/s13205-020-02407-x

**Published:** 2020-08-28

**Authors:** Aido Taloh, D. V. S. Raju, Namita Banyal, Gunjeet Kumar, Priyam Panda, R. Manimekalai, Carmine Marcone, G. P. Rao

**Affiliations:** 1 Division of Floriculture and Landscaping, ICAR-Indian Agricultural Research Institute, New Delhi, 110012 India; 2 Directorate of Floricultural Research, ICAR-College of Agriculture Campus Shivajinagar, Pune, 411005 India; 3 Discipline of Life Sciences, Indira Gandhi National Open University, New Delhi, 110068 India; 4 Department of Pharmacy, University of Salerno, 84084 Fisciano, Salerno Italy; 5 Division of Plant Pathology, ICAR-Indian Agricultural Research Institute, New Delhi, 110012 India; 6 Sugarcane Breeding Institute, Coimbatore, Tamil Nadu 671003 India

**Keywords:** *Chrysanthemum morifolium*, Phytoplasma ribosomal subgroups, Virtual RFLP anlaysis

## Abstract

Symptoms typical of phytoplasma infection such as phyllody, virescence, witches’ broom and yellowing were observed in 12 varieties of *Chrysanthemum morifolium* in floral nurseries and experimental fields at New Delhi, Karnataka, Maharashtra and Andhra Pradesh, India, during surveys made from 2015 to 2017. Disease incidence ranged from 15 to 30%. Phytoplasma presence was confirmed in all symptomatic chrysanthemum varieties by molecular identification assays. Sequence comparison, phylogenetic and in silico RFLP analyses of 16S rDNA sequences allowed the identification of the chrysanthemum infecting phytoplasma strains into different ribosomal groups and subgroups, namely 16SrI, 16SrII-D, 16SrVI-D and 16SrXIV. Detection of phytoplasma strains of 16SrII-D subgroup were also confirmed in symptomatic *Chenopodium album* and *Parthenium hysterophorus* plants grown in and around the surveyed chrysanthemum fields at New Delhi, whereas 16SrVI-D phytoplasma strains were detected in symptomatic *Cannabis sativa* weed and leafhopper *Hishimonus phycitis* individuals collected from the symptomatic chrysanthemum fields at New Delhi. This is the first report on the presence of 16SrVI and 16SrXIV groups of phytoplasmas in chrysanthemum plants. Studies on genetic diversity of phytoplasmas infecting the major chrysanthemum varieties in India and their epidemiological aspects had previously not been reported. The detection and identification of phytoplasmas in different chrysanthemum varieties could contribute to increase the awareness among farmers in the management of these diseases.

## Introduction

Chrysanthemum (*Chrysanthemum morifolium* Ramat) occupies 3rd and 5th positions in the cut flower and pot plant trades; respectively (Anonymous 2015). It is used as cut flower and loose flower due to its attractive form and colour of flower. In India, it is grown commercially on a large area of 16.63 thousand ha with a production of 186.06 thousand MT (Anonymous 2014). Flower crops are affected worldwide by several biotic and abiotic stresses and phytoplasma-associated diseases are the major threat to commercial cultivations and are responsible of severe economic losses (Chaturvedi et al. 2010; Bellardi et al. 2018).

The occurrence of phytoplasmas in chrysanthemum has been reported from Japan, Italy, China, Iran and India (Shiomi and Sugiura 1983; Conti et al. 1988; Raj et al. 2007; Chung 2008; Min et al. 2009; Rani et al. 2014; Yadav et al. 2015). Although chrysanthemum has been grown at a commercial scale in different states of India, very little is known about the phytoplasma strains infecting this crop (Rao et al. 2017). Therefore, the genetic diversity of phytoplasmas infecting the major chrysanthemum varieties in India was studied. In addition, molecular identification of these phytoplasmas presence in weeds that may act as alternative hosts and potential insect vectors responsible of their spread were also made.

## Materials and methods

**Fig. 1:**
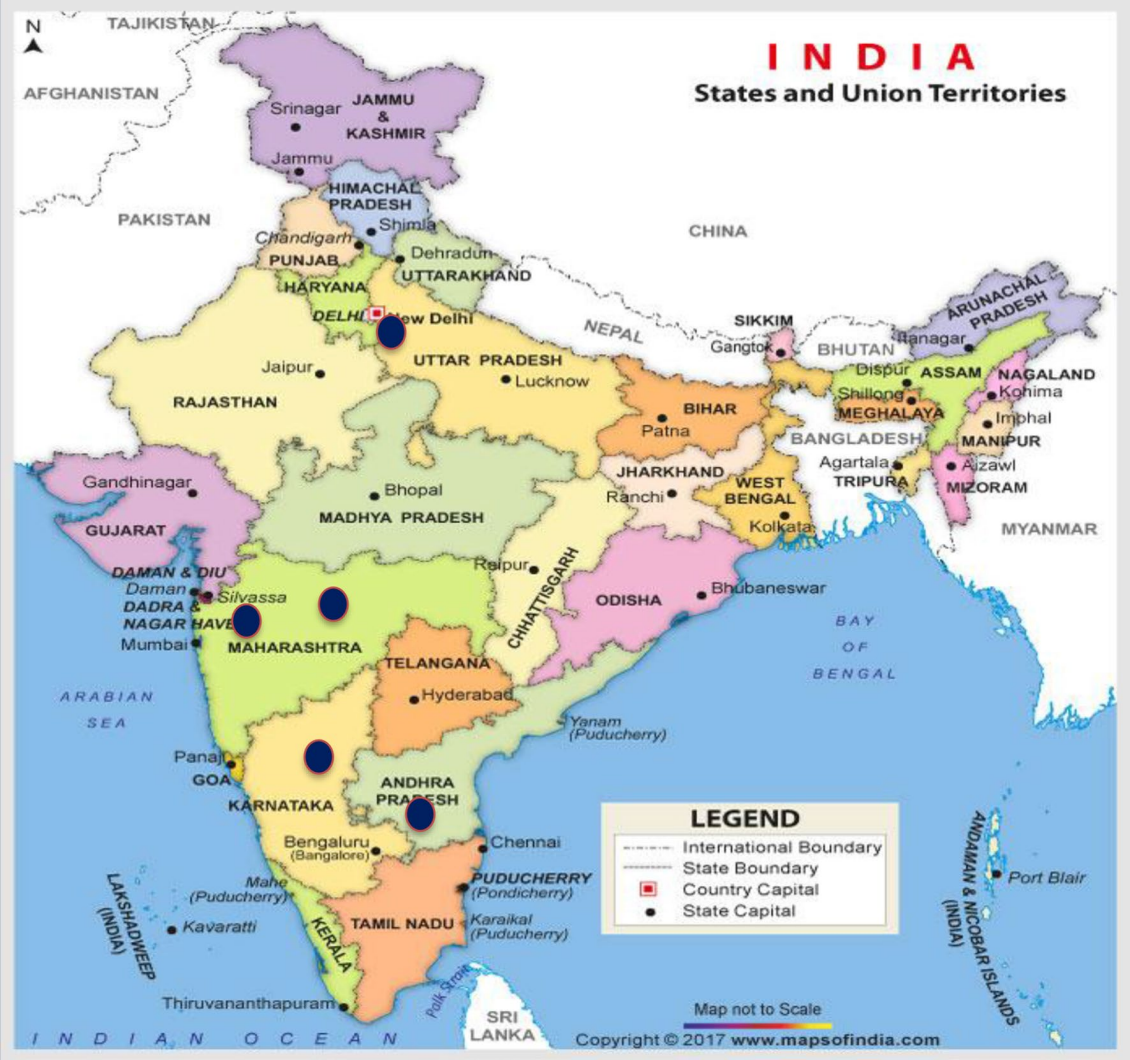
Map of India showing the survey locations in the present study

Surveys of gardens, nurseries and experimental fields at ICAR-Indian Agricultural Research Institute (ICAR-IARI), New Delhi, ICAR-Indian Institute of Horticultural Research (IIHR), Bangalore, Karnataka, Maharashtra (Bandra and Pune) and Andhra Pradesh (Rajahmundry) were made from October 2015 to February 2017 (Fig. 1). Symptoms of phytoplasma infections were recorded on 12 chrysanthemum varieties from 5 surveyed locations. The disease incidence on each variety was evaluated on the basis of visual inspection by counting the number of symptomatic plants in relation to the total number of plants inspected using formula:

$$ {\text{Percentage of disease incidence }} = \frac{{{\text{No}}.{\text{ of symptomatic plants for each variety}} }}{{{\text{Total no}}.{\text{ of plants inspected for each variety}}}} \times { 1}00 $$

Three leaf samples each of symptomatic chrysanthemum varieties from all the four locations and three weed species (*Chenopodium album*, *Parthenium hysterophorus* and *Cannabis sativa*) from IARI, New Delhi were collected (Table 1). Toria phyllody phytoplasma (Azadvar et al. 2009) maintained in periwinkle (*Catharanthus roseus*) in the greenhouse was used as a positive control.

**Table 1:** Detection and identification of phytoplasmas occurring in chrysanthemum varieties, weeds and leafhoppers in different locations in India

Sr. No	Locations, chrysanthemum varieties and weeds	Symptoms	Average incidence* (%)	Survey period	GenBank Acc. No	Phytoplasma ribosomal group/subgroup
	**IARI, New Delhi**					
	**Chrysanthemum varieties**					
	Pusa Centenary	Severe witches’ broom and virescence	16.8	October 2015	KY612250	16SrII-D
	Pusa Kesari	Phyllody and virescence	20.0	October 2015	KX641015	16SrI
	Ajay Orange strain 1 strain 2	Severe phyllody and witches’ broom	29.6 27.5	November 2015 February 2016	KX641012 KY693690	16SrII-D 16SrII-D
	Pusa Anmol Strain 1 Strain 2	Severe virescence and phyllody	30.0 28.3	November 2015 March 2016	KY693687 KY693688	16SrII-D 16SrII-D
	Red Spoon	Witches’ broom and phyllody	25.0	March 2016	MF040140	16SrII-D
	Jaya	Phyllody	22.5	April 2016	KY693689	16SrVI-D
	Johan Webber	Phyllody	17.5	May 2016	KX641014	16SrVI
	**Weeds**					
	*Chenopodium album* (Amaranthaceae)	Witches’ broom	12.6	April 2015	MF040139	16SrII-D
	*Cannabis sativa* (Cannabinaceae)	Witches’ broom	11.2	May 2016	MF509775	16SrVI-D
	*Parthenium hysterophorus* (Asteraceae)	Phyllody and witches’ broom	14.5	August 2016	MF040141	16SrII-D
	**Insect**					
	*Hishimonus phycitis*	Cicadellidae	–	March 2016	KY856746	16SrVI-D
1	**Maharashtra (DFR, Pune)**					
	White Nursery	Leaf yellowing and stunting	23.3	March 2015	KY693691	16SrVI-D
	Nayak Yellow	Leaf curling and yellowing	26.6	March 2015	KX641016	16SrXIV
2	**Bandra, Mumbai** Meera	Phyllody	20	November 2016	KY472314	16SrI
3	**Karnataka (IIHR, Bangalore)**					
	Yellow Gold	Necrosis and witches’ broom	20	November 2016	KY693692	16SrI
4	**Andhra Pradesh (Kadiam, Rajahmundary)**					
	Indira	Leaf yellowing and severe stunting	15	December 2016	KY693693	16SrII

* Average incidence calculated on the basis of visual observation of symptoms in different fields

Asymptomatic chrysanthemum plant samples were collected from the fields of the same area but at distance from the symptomatic fields with no symptoms and used them as negative controls. Leafhopper species from the surveyed chrysanthemum fields at New Delhi were also collected at 15 days intervals from October 2016 to February 2017 by the sweeping net method. The captured leafhoppers were identified by morphology as per taxonomy manual on identification of leafhoppers (Viraktamath and Meshram 2019) at Division of Entomology, ICAR- Indian Agricultural Research Institute, New Delhi.

DNA from plants and insects was extracted employing CTAB-based extraction procedures as described (Ahrens and Seemüller 1992; Maixner et al. 1995). PCR amplification was performed with the universal phytoplasma primer pair P1/P7 (Deng and Hiruki 1991; Schneider et al. 1995) followed by primer pair 3Far/3Rev (Manimekalai et al. 2010). PCR reactions were carried out following the protocol described by Rao et al. (2014). Five microlitres of PCR product was subjected to electrophoresis in a 1.0% (w/v) agarose gel, stained with ethidium bromide and observed under a UV transilluminator. All PCR products (~ 1.3 kb) were purified using the Wizard^®^ SV Gel and PCR Clean- up System (Promega, Madison, USA).

PCR products were ligated in pGEM^®^T vector (Promega) and cloned in *Escherichia coli* (DH5-α) following the manufacturer’s instructions, the cloned products were outsourced for sequencing using M13Fwd/M13Rev universal primer pair in both directions at Agrigenome, Kerala, India. The sequences were assembled using DNA baser V.4 software online tool and submitted to GenBank. A database search was performed by BLASTn analysis at NCBI (www.ncbi.org). The 16S rRNA gene sequences were aligned with phytoplasma group/subgroup representatives available in GenBank using ClustalW (Thompson et al. 1994) and used to construct a phylogenetic tree by the Neighbor-joining method with 1000 replications for each bootstrap value using MEGA 7.0 software (Kumar et al. 2016). The 16Sr DNA sequence (Acc. No. AB680603) of *Acholeplasma laidlawii* was used as out group to root the phylogenetic tree.

## Results

**Fig. 2:**
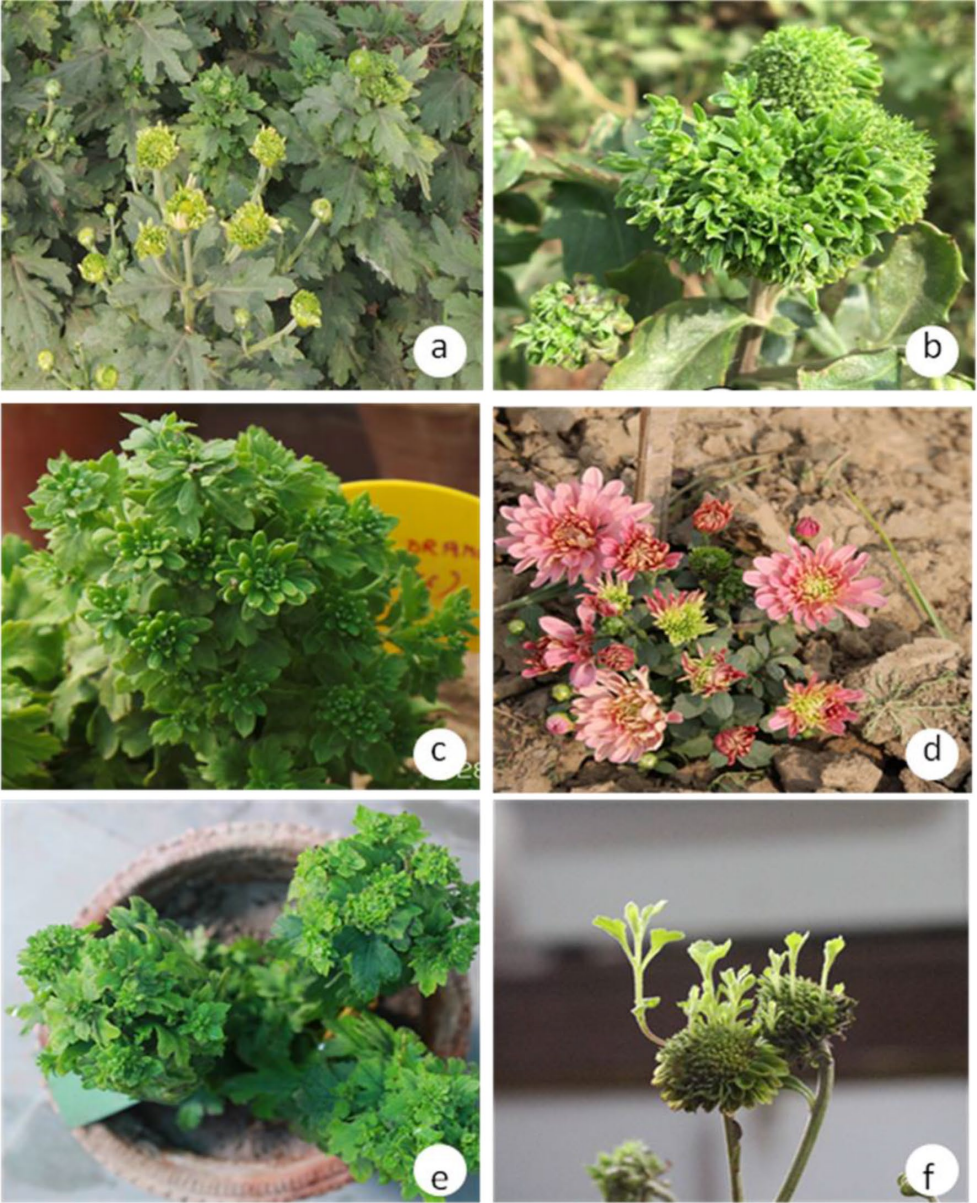
Phytoplasma disease symptoms in different chrysanthemum varieties at IARI, New Delhi: **a** PusaKesari: phyllody and virescence symptoms; **b** Pusa centenary: witches’ broom and flower virescence symptoms; **c** Ajay Orange: severe phyllody and witches’ broom; **d** Pusa Anmol: severe virescence and phyllody; **e** Red Spoon: witches’ broom and phyllody; **f** Jaya: phyllody

**Fig. 3:**
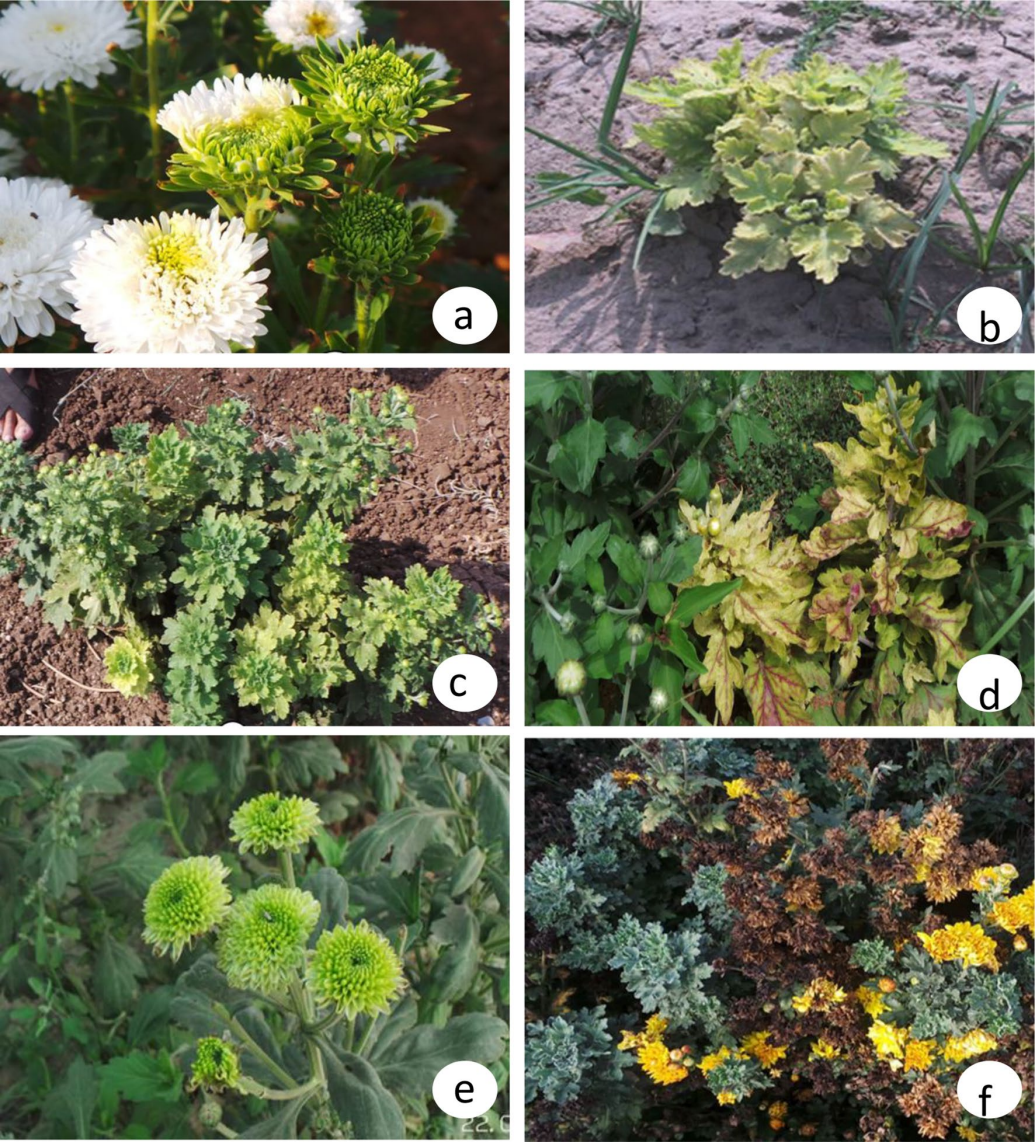
Phytoplasma disease symptoms in different chrysanthemum varieties **a** Johan Webber: severe phyllody at IARI New Delhi; **b** Indira: leaf yellowing and severe stunting at Kadiam, Rajahmundry; **c** White Nursery: yellowing and stunting at DFR, Pune; **d** Nayak Yellow: leaf curling and yellowing at DFR, Pune; **e** Meera: greening of flowers at Bandra; **f** Yellow Gold: necrosis and witches’ broom at IIHR, Bangalore

During survey of gardens, nurseries, experimental fields in four states of India, symptoms of leaf yellowing, phyllody, virescence, witches’ broom and stunting were recorded on chrysanthemum varieties. The phyllody and witches’ broom symptoms were recorded as most common symptoms (Figs. 2, 3). The symptoms recorded on chrysanthemum varieties in four states of India are listed in Table 1. The disease incidence on different chrysanthemum varieties were recorded from 15% in Indira variety at Kadiam, Rajahmundry, Andhra Pradesh to 30% in Pusa Anmol variety at IARI, New Delhi (Table 1). Symptoms were most pronounced at the flowering stage at all the surveyed locations.

**Fig. 4:**
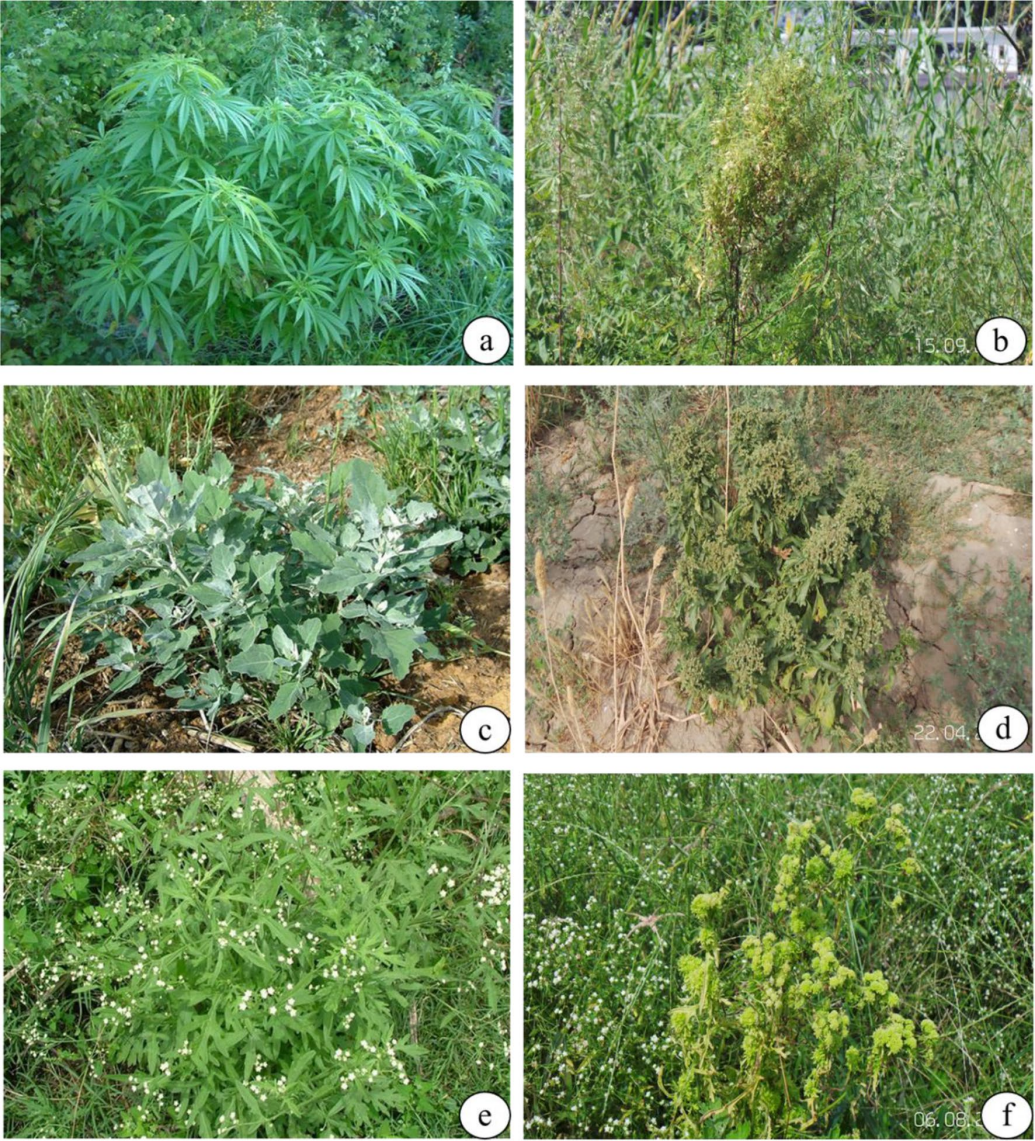
Phytoplasma disease symptoms in different weed species at IARI, New Delhi: **a** *Cannabis sativa* (Healthy); **b** *Cannabis sativa* (Infected); **c** *Chenopodium album* (Healthy); **d** *Chenopodium album* (Infected); **e** *Parthenium hysterophorus* (Healthy); **f** *Parthenium hysterophorus* (Infected)

Symptoms including leaf chlorosis, phyllody and witches’ broom were also observed in *Chenopodium album*, *Parthenium hysterophorus* and *Cannabis sativa* grown in and around the chrysanthemum fields at ICAR-IARI, New Delhi (Table 1, Fig. 4). Among the weed species, *P. hysterophorus* showed the highest disease incidence (14.5%) followed by *C*. *album* (12.60%) and *C*. *sativa* (11.20%) (Table 1).

All the symptomatic chrysanthemum and weed plants (Table 1) along with toria phyllody phytoplasma strain yielded amplicons of ~ 1.3 kb in nested PCR assays. No amplification products were obtained from the template DNA from any asymptomatic plants with each of primer pair employed. Leafhoppers collected from the surveyed chrysanthemum experimental fields at IARI, New Delhi were identified as *Hishimonus phycitis*, *Empoasca motti*, *Hecalus porrectus*, *Amrasca biguttula* and *Psammotettix* spp. Among these species, *H. phycitis* was recorded as the most abundant species.

Amplification products were obtained from individuals of *H. phycitis* but not from those of the other four leafhopper species (*Empoasca motti*, *Hecalus porrectus*, *Amrasca biguttula, Psammotettix* spp) collected from the field at IARI, New Delhi (data not shown).

A BLASTn identity search of GenBank database revealed that phytoplasma strains infecting chrysanthemum plants of varieties Pusa Centenary (GenBank Acc. No.KY612250), Ajay Orange (GenBank Acc. Nos. KX641012, KY693690), Pusa Anmol (GenBank Acc. Nos. KY693687, KY693688), Red Spoon (GenBank Acc. No. MF040140) and Indira from Rajahmundry, Andhra Pradesh (GenBank Acc. No.KY693693), all from IARI, New Delhi showed 16S rDNA sequence similarity 98.5 to 100% with 16SrII phytoplasma group (Table 1). Phytoplasma strains detected in chrysanthemum plants of varieties Jaya (GenBank Acc. No. KY693689) and Johan Webber (GenBank Acc. No. KX641014), both from IARI, New Delhi, and White Nursery (GenBank Acc. No. KY693691) from DFR, Pune showed 16S rDNA sequence similarity 99.4–99.8% with ‘*Candidatus* Phytoplasma trifolii’ 16SrVI group. However, the phytoplasma strains associated with Pusa Kesari from IARI, New Delhi (GenBank Acc. No. KX641015), Yellow Gold from IIHR, Bangalore (GenBank Acc. No.KY693692), and Meera from Bandra, Mumbai (GenBank Acc. No. KY472314) showed sequence similarity ranging from 99.7 to 100% with 16SrI group (Table 1), whereas phytoplasma strains from chrysanthemum Nayak Yellow (GenBank Acc. No. KX641016) from IARI, New Delhi, hosted a phytoplasma strain which showed 16S rDNA sequence similarity 99.6% with 16SrXIV group (Table 1).

Phytoplasma strains detected in *C. album* (GenBank Acc. No. MF040139) and *P. hysterophorus* (GenBank Acc. No.MF040141) plants showed a 99.9–100% 16S rDNA sequence identity with phytoplasma described in peanut witches’ broom group whereas those present in *C. sativa* plants and *H. phycitis* leafhopper showed a 99.9–100% 16S rDNA sequence identity with 16SrVI group.

**Fig. 5:**
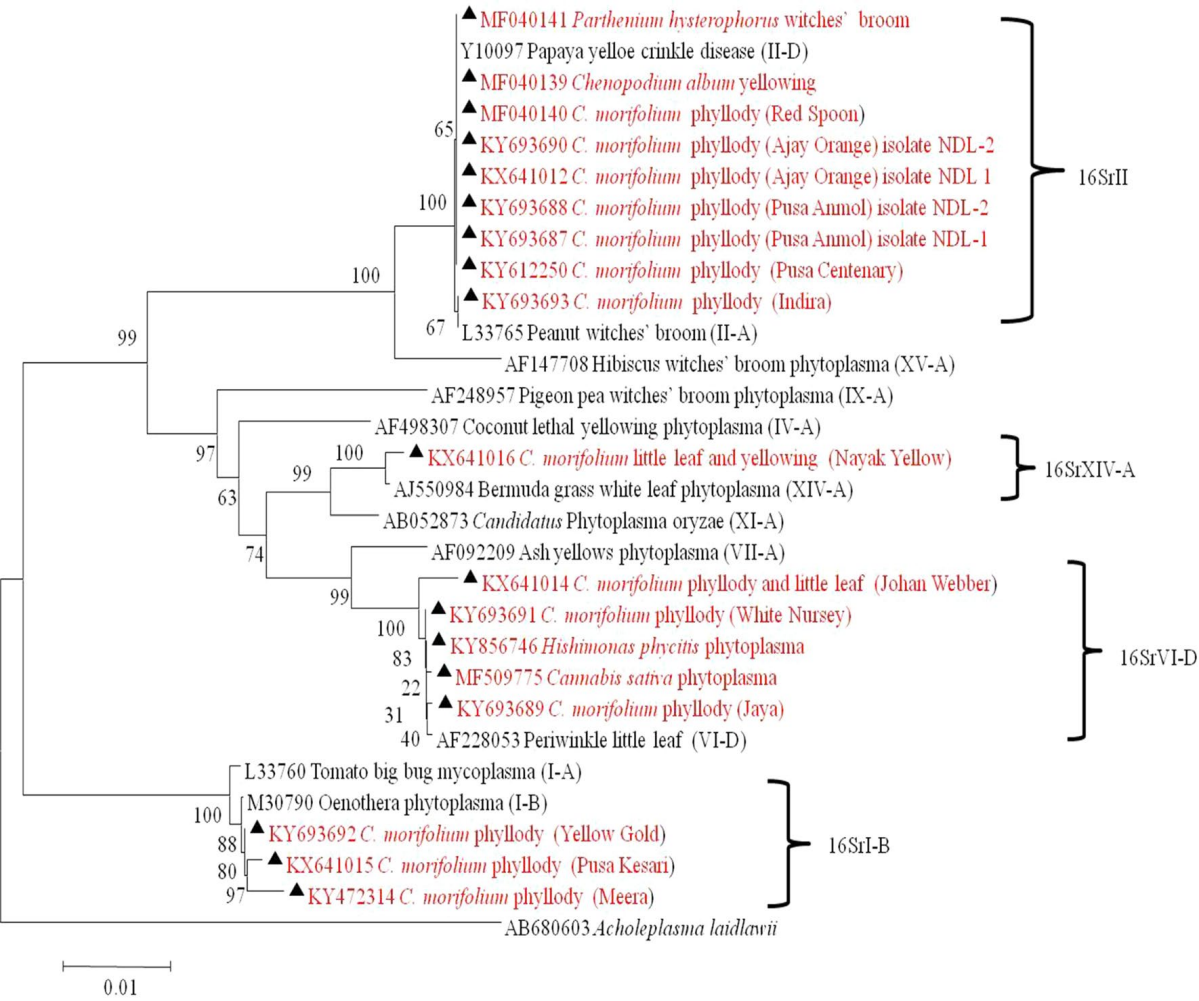
Phylogenetic tree based on 16Sr DNA constructed by neighbor-joining method showing the relationship among chrysanthemum varieties, weed species and insect phytoplasma strains, and reference phytoplasma strains. Accession numbers are specified in the tree*. ‘Acholeplasma laidlawaii’* was used as outgroup. Mega 7.0 software was used to construct the tree. Numbers on branches are bootstrap values obtained for 1000 replicates. The bar represents a phylogenetic distance of 0.01

**Fig. 6:**
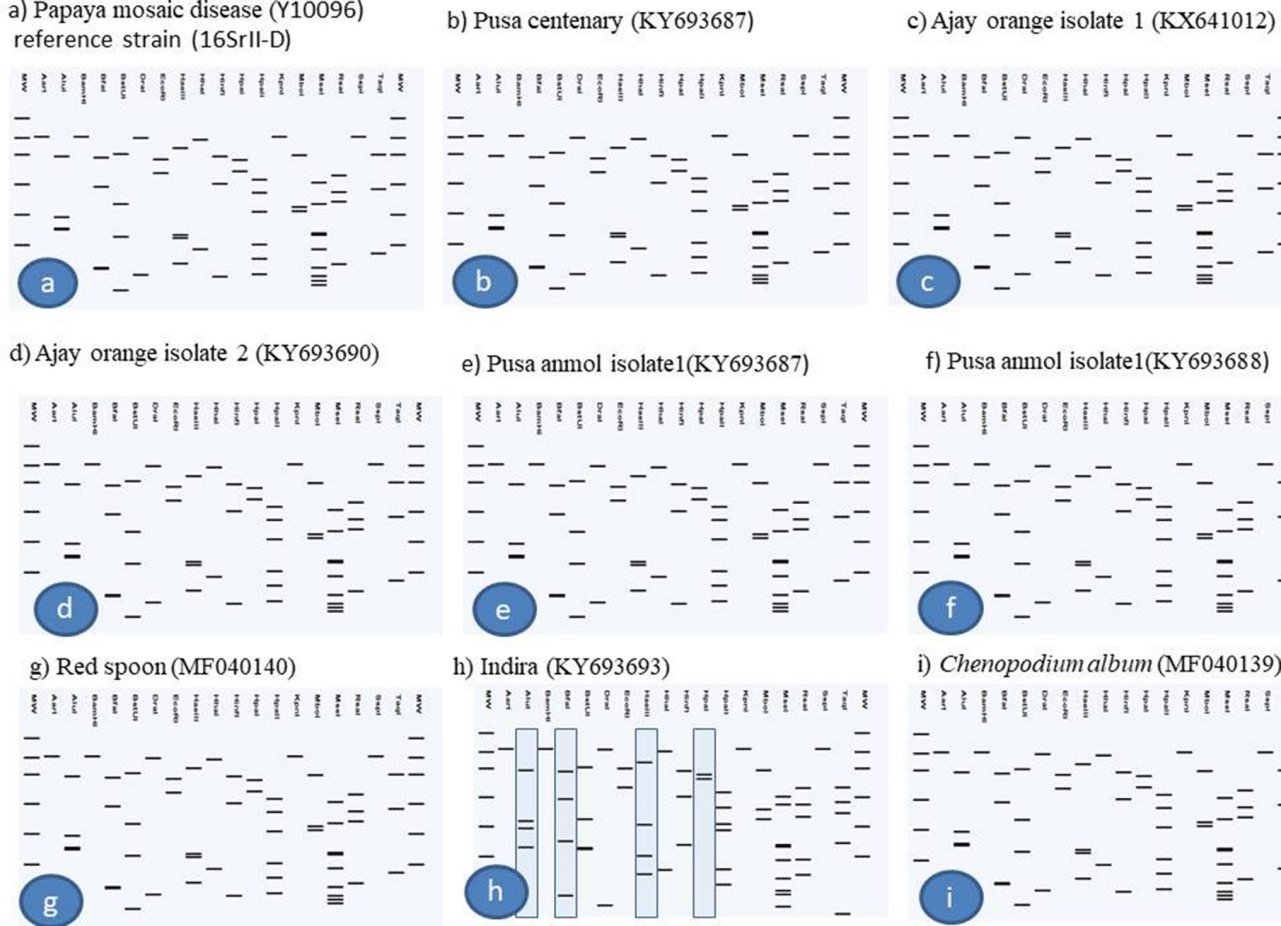
Comparison of virtual RFLP patterns derived from in silico digestions of ~ 1.3 kb 16S rDNA sequences of **a** Reference strain 16SrII-D Mollicutes sp. (associated with papaya mosaic disease) 16S rRNA gene (GenBank Acc. No. Y10096); **b** Pusa Centenary (GenBank Acc. No. KY612250); **c** Ajay orange strain 1 (GenBank Acc. No. KY641012); **d** Ajay orange strain 2 (GenBank Acc. No. KY693690; **e** Pusa anmol strain1(GenBank Acc. No KY693687; **f** Pusa anmol strain 1(GenBank Acc. No KY693688; **g** Red Spoon (GenBank Acc. No. MF040140); **h** Indira (GenBank Acc. KY693693); **i** *Chenopodium album* (GenBank Acc No. MF040139); **j** *Parthenium hysterophorus* (GenBank Acc. No. MF040141) digested using 17 different restriction endonucleases indicating that chrysanthemum varieties phytoplasma belonged to **16SrII-D** phytoplasma sub groups, but the Indira strain showed variation for the RFLP pattern

**Fig. 7:**
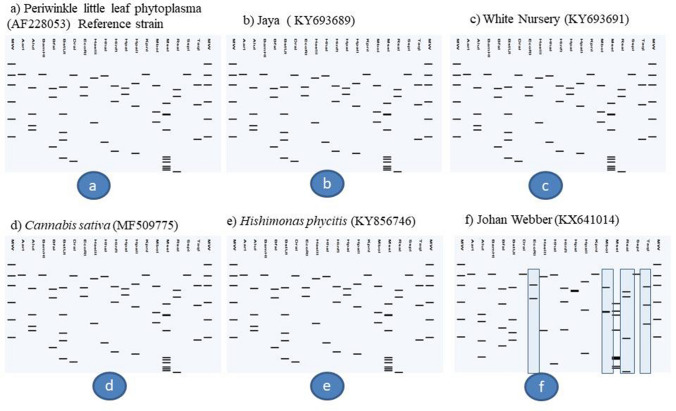
Comparison of virtual RFLP patterns derived from in silico digestions of ~ 1.3 kb 16S rDNA sequences of **a** Reference strain 16SrVI-D Periwinkle little leaf phytoplasma 16S rRNA gene. (GenBank Acc. No. AF228053), **b** Jaya (GenBank Acc. No. KY693689) **c** White Nursery (GenBank Acc. No. KY693691, **d** *Cannabis sativa* (GenBank Acc. No. MF509775) **e** *Hishimonas phycitis* phytoplasma (GenBank Acc. No KY856746) and **f** Johan Webber (GenBank Acc. No. KX641014) digested using 17 different restriction endonucleases indicating that Jaya, White Nursery, *Cannabis sativa* and *Hishimonas phycitis* phytoplasma belonged to **16SrVI-D** sub groups, but Johan Webber 'Chrysanthemum showed variation for RFLP pattern and could not be assigned subgroup

**Fig. 8:**
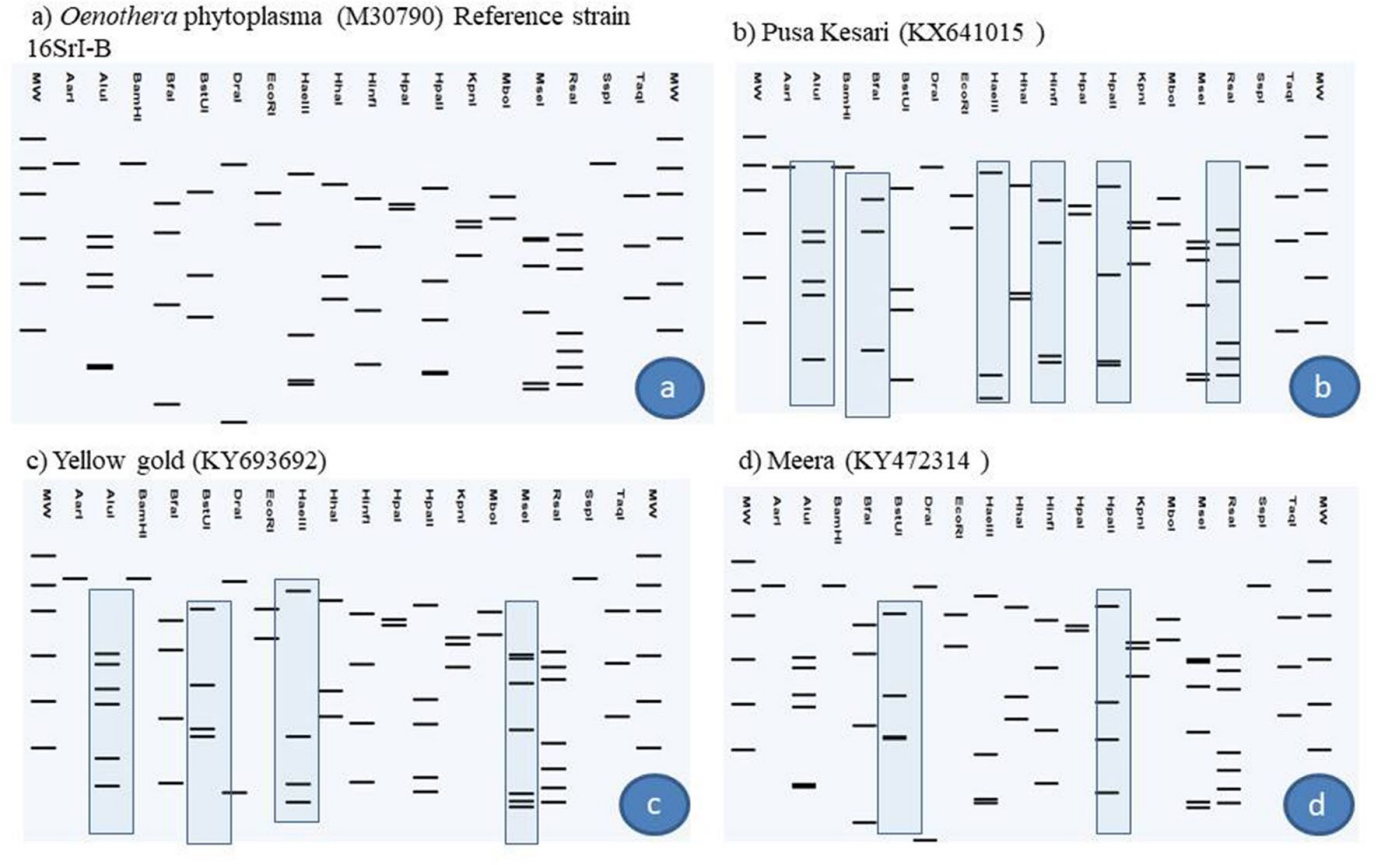
Comparison of virtual RFLP patterns derived from in silico digestions of ~ 1.3 kb 16S rDNA sequences of **a** Reference strain *Oenothera* phytoplasma (GenBank Acc. No. M30790) and **b** Pusa Kesari phytoplasma (GenBank Acc. No. KX641015) **c** Yellow gold phytoplasma (GenBank Acc. No. KY693692) and **d** Meera phytoplasma (GenBank Acc. No. KY472314) digested using 17 different restriction endonucleases indicating variations for RFLP pattern. Hence subgroup could not be assigned

**Fig. 9:**
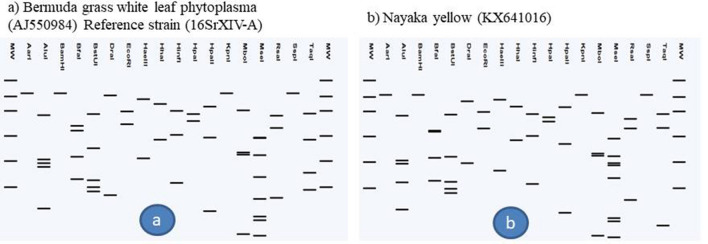
Comparison of virtual RFLP patterns derived from in silico digestions of ~ 1.3 kb 16S rDNA sequences of **a** Reference strain 16SrXIV-A, Bermuda grass white leaf phytoplasma 16S rRNA gene (GenBank Acc. No. AJ550984), **b** Nayaka yellow (GenBank Acc. No. KX641016) digested using 17 different restriction endonucleases indicating that this strain belonged to 16SrXIV group but could not be assigned subgroup 16SrXIV-A subgroup because of difference in MseI and Taq1 restriction profiles

Phylogenetic analysis of 16S rDNA sequences showed that phytoplasma strains detected in different varieties of chrysanthemum, weed plants and *H. phycitis* leafhoppers were clustered with phytoplasmas enclosed in 16SrI, 16SrII, 16SrVI and 16SrXIV groups (Fig. 5). Subgroup assignment by comparison of virtual RFLP patterns derived from in silico digestions 17 restriction endonucleases indicated Pusa Centenary (GenBank Acc. No. KY612250), Ajay orange strain 1 (GenBank Acc. No. KX641012) Ajay orange strain 2 (GenBank Acc. No. KY693690), Pusa anmol strain1 (GenBank Acc. No KY693687) and Pusa anmol strain 2 (GenBank Acc. No KY693688) Red spoon (GenBank Acc. No. MF040140), *Chenopodium album* (GenBank Acc. No. MF040139) and *Parthenium hysterophorus* (GenBank Acc. No. MF040141) belonged to 16SrII subgroup-D, However the RFLP pattern of phytoplasma strain Indira (GenBank Acc. No. KY693693) with the reference strain papaya mosaic disease phytoplasma (GenBank Acc. No. Y10096) showed variation for enzymes *Alu*I, *Bfa*I, *Hae*III and *Hpa*I could not be assigned to subgroups because of variation in the RFLP patterns with reference strain (Fig. 6a, Table 1). Jaya (GenBank Acc. No. KY693689), White Nursery (GenBank Acc. No. KY693691), *Cannabis sativa* (GenBank Acc. No. MF509775) and *H. phycitis* phytoplasma strain (GenBank Acc. No KY856746) belonged to 16SrVI subgroup-D. However, the RFLP pattern of phytoplasma strain Johan webber (GenBank Acc. No. KX641014) with the reference strain periwinkle little leaf phytoplasma (GenBank Acc. No. AF228053) showed variation for enzymes *EcoR*I, *Mbo*I, *Rsa*I and *Taq*I and could not be assigned to subgroups because of variation in the RFLP patterns with reference strain of 16SrVI-D subgroup (Fig. 7, Table 1). The RFLP pattern of phytoplasma strain Pusa Kesari (GenBank Acc. No. KX641015) showed variation for enzymes *Alu*I, *BstU*I, *Hae*III and *Mse*I; Yellow gold (GenBank Acc. No. KY693692) showed variation for enzymes *Alu*I, *Bfa*I, *Hae*III, *Hlnf*I, *Hpa*II and *Rsa*I and Meera (GenBank Acc. No KY472314) showed variation for enzymes, *BstU*I and *Hpa*II with reference strain of 16SrI-B subgroup (GenBank Acc. No. M30790) could not be assigned to subgroups because of variation in the RFLP patterns with reference strain (Fig. 8, Table 1). The Nayaka yellow (GenBank Acc. No. KX641016) belonged to 16SrXIV group but could not assigned to subgroup because of variation in profilings of *Mse*1 and *Taq*1 (Fig. 9, Table 1).

## Discussion

Different groups of phytoplasmas are reported earlier with different chrysanthemum species. Symptoms induced by aster yellows phytoplasma infection included stunt, yellowing, virescence and phyllody in *C. frutescence* in Italy (Bertaccini et al. 1992; Conti et al. 1988). The presence of phytoplasmas affiliated to aster yellows group in chrysanthemum was reported earlier from Italy, Korea and India (Marzachi and Bosco 2005; Chung 2008; Raj et al. 2007). Chrysanthemum yellows phytoplasma (16SrI-B subgroup) associated with witches’ broom, vein clearing, dwarf and abnormal production of secondary shoots was reported in *C. carinatum* and *C. morifolium* from European and Mediterranean areas (Bertaccini et al. 1990; Conti et al. 1988; Saracco et al. 2005). The identification of 16SrII group phytoplasmas in *C. grandiflorum* was reported in Okinawa Prefecture, Japan (Naito et al. 2007) and India (Yadav et al. 2015). Min et al. (2009) reported a new outbreak of phytoplasma disease of chrysanthemum with symptoms of yellowing of leaf margins, flattened stem and shortening of internodes in China associated with 16SrI group, which was responsible for serious losses in flower quality.

Limited reports are available on identification of phytoplasma strains association with chrysanthemum varieties in India (Raj et al. 2007; Yadav et al. 2015). However, in the present study, two subgroups belonging to four groups of phytoplasmas (16SrI, 16SrII-D, 16SrVI-D and 16SrXIV) infecting 12 varieties of chrysanthemum were identified from four major chrysanthemum growing states of India. However, the restriction profiles of phytoplasma strain associated with chrysanthemum variety Johan weber from Delhi (Acc. No. KX641014), Nayak yellow from Pune (Acc. No. KX641016), Meera from Mumbai (Acc. No. KY472314) and Indira from Andhra Pradesh did not match completely with any of the earlier classified subgroups, hence they may be new strains and multilocus genes specific detection and real RFLP analysis are necessary to confirm the reported unusual variability.

Several weeds are reported as reservoirs of important phytoplasma strains which are suggested to play an important role in natural spread of phytoplasma strains and serve as natural alternative hosts, since they influence the population density of the vectors and act as source of inoculums (Pasquini et al. 2007; Duduk et al. 2018). In the present study, two phytoplasmas ribosomal groups (16SrII and 16SrVI) were identified in three weed species, that are also reported to be reservoir of different phytoplasma strains in different countries, viz., 16SrII-E subgroup in Italy (Tolu et al. 2006), 16SrIII (X-disease) group and 16SrXII group in Czech Republic (Safarova et al. 2011). In the present study, the report of *Chenopodium* sp. as a host of 16SrII group indicates its potentiality as natural reservoir. Earlier, *P. hysterophorus* weed was also reported as host of 16SrI and 16SrII group in India and China (Raj et al. 2009; Li et al. 2011; Mall et al. 2015; Yadav et al. 2016). Further, *C. sativa* and *Achyranthes aspera* weed species were also reported as host of 16SrI, 16SrII, 16SrVI and 16SrXIV groups of phytoplasmas in India (Rao et al. 2017), which suggests potentiality of these weed species as natural reservoirs of phytoplasmas. *C. album, P. hysterophorus* and *C. sativa* weed species that are prevalent in chrysanthemum fields at New Delhi and resulted positive for the 16SrII and 16SrVI groups of phytoplasmas in the present study may also play an important role for natural spread of phytoplasma strains.

Limited information is available on transmission of phytoplasma strains associated with ornamentals through insect vectors. Bosco et al. (2007) reported transmission of chrysanthemum yellows phytoplasma through three leafhopper vector species (*Euscelis incisus*, *Euscelidius variegatus* and *Macrosteles quadripunctulatus*) in Italy. *Empoasca decipiens* was proved to be an experimental vector in transmitting chrysanthemum yellows phytoplasma (16SrI) in Italy (Galetto et al. 2011a; b)*.* However, in the present study, the leafhopper, *H*. *phycitis* was positive to16SrVI group phytoplasmas. The identification of 16SrVI group of phytoplasma strain in *H. phycitis* and in two chrysanthemum varieties, Jaya and Johan Webber, in same field suggest it as vector, which needs further confirmation through transmission assays. This leafhopper species has already been reported as vectors for 16SrI and 16SrII phytoplasma groups in India (Nabi et al. 2015; Gopala and Rao 2018), which indicates its potentiality to transmit different phytoplasma strains. In India, many crops are being cultivated in parallel with other agricultural crops in different seasons. The widespread occurrence of phytoplasma strains identified in chrysanthemum varieties in the study pose a serious threat of chrysanthemum cultivation in India and needs further studies on epidemiology and management.

## References

[CR1] Ahrens U, Seemüller E (1992). Detection of DNA of plant pathogenic mycoplasma like organisms by a polymerase chain reaction that amplifies a sequence of the 16S rRNA gene. Phytopathology.

[CR2] Anonymous (2014) NHB database. Indian Horticulture Database, Gurgaon, Haryana. Ministry of Agriculture, Government of India. https://nhb.gov.in

[CR3] Anonymous (2015) Flora Holland.Legmeerdijk 313, Aalsmeer, Netherlands. https://www.floraholland.com

[CR4] Azadvar M, Baranwal VK, Yadava DK (2009). First report of a 16SrIX (Pigeon pea witches’ broom) phytoplasma associated with toria (*Brassica rapa* cv. toria) phyllody disease in India. New Dis Rep.

[CR5] Bellardi MG, Betaccini A, Madhupriya RGP, Rao GP, Bertaccini A, Fiore N, Liefting LW (2018). Phytoplasma disease in ornamental crops. Phytoplasmas: plant pathogenic bacteria-I, characterization and epidemiology of phytoplasma-associated diseases.

[CR6] Bertaccini A, Davis RE, Lee I-M, Conti M, Dally EL, Douglas SM (1990). Detection of chrysanthemum yellows mycoplasma like organism by dot hybridization and Southern blot analysis. Plant Dis.

[CR7] Bertaccini A, Davis RE, Hammond RW, Bellardi MG, Vibio M, Lee IM (1992). Sensitive detection of mycoplasma like organisms in field collected and *in vitro* propagated plants of *Brassica*, *Hydrangea* and *Chrysanthemum* by polymerase chain reaction. Ann Appl Biol.

[CR8] Bosco D, Galetto L, Leoncini P, Saracco P, Raccah B, Marzachì C (2007). Interrelationships between ‘*Candidatus*Phytoplasma asteris’ and its leafhopper vectors (Homoptera: Cicadellidae). Econ Entomol.

[CR9] Chaturvedi Y, Singh M, Snehi SK, Raj SK, Rao GP (2010). First report of ‘*Candidatus* Phytoplasma asteris’ (16Sr I group) associated with yellows and little leaf diseases of *Hibiscus rosa*-*sinensis* in India. Plant Pathol.

[CR10] Chung BN, Harrison NA, Rao GP, Marcone C (2008). Phytoplasma detection in Chrysanthemum and lily. Characterization, diagnosis and management of phytoplasma.

[CR11] Conti M, Agostino GD, Casetta A, Mela L (1988). Some characteristics of Chrysanthemum yellows disease. Acta Hortic.

[CR12] Deng S, Hiruki C (1991). Amplification of *16S rRNA* genes from culturable and nonculturable mollicutes. J Microbiol Methods.

[CR13] Duduk B, Stepanovi`c J, Yadav A, Rao GP, Rao GP, Bertaccini A, Fiore N, Liefting LW (2018). Phytoplasma in weeds and wild plants. Phytoplasmas: plant pathogenic bacteria-I, characterisation and epidemiology of phytoplasma-associated diseases.

[CR14] Galetto L, Bosco D, Balestrini R, Genre A, Fletcher J, Marzachì C (2011). The major antigenic membrane protein of “*Candidatus* Phytoplasma asteris” selectively interacts with ATP synthase and actin of leafhopper vectors. PLoS ONE.

[CR15] Galetto L, Marzachì C, Demichelis S, Bosco D (2011). Host plant determines the phytoplasma transmission competence of *Empoasca decipiens* (Hemiptera: Cicadellidae). J Econ Entomol.

[CR16] Gopala, Rao GP (2018). Molecular characterization of phytoplasma associated with four important ornamental plant species in India and identification of natural potential spread sources. 3 Biotech.

[CR18] Kumar S, Stecher G, Tamura K (2016). MEGA 7: molecular evolutionary genetics analysis version 7.0 for bigger datasets. Mol Biol Evol.

[CR19] Li Z, Zhang L, Che H, Liu H, Chi M, Luo D, Li Y, Chen W, Wu Y (2011). A disease associated with phytoplasma in *Parthenium hysterophorus*. Phytoparasitica.

[CR20] Maixner M, Ahrens U, Seemüller E (1995). Detection of the German grapevine yellows (Vergilbungskrankheit) MLO in grapevine, alternative hosts and a vector by a specific PCR procedure. Eur J Plant Pathol.

[CR21] Mall S, Kumar S, Jadon VJ, Rao GP (2015). Identification of phytoplasmas associated with weed species in India. Indian Phytopathol.

[CR22] Manimekalai R, Soumya VP, Sathish KR, Selvarajan R, Reddy K, Thomas GV, Sasikala M, Rajeev G, Baranwal VK (2010). Molecular detection of 16SrXI group phytoplasma associated with root (wilt) disease of coconut (*Cocos nucifera*) in India. Plant Dis.

[CR23] Marzachí C, Bosco D (2005). Relative quantification of chrysanthemum yellows (16SrI) phytoplasma in its plant and insect host using real-time polymerase chain reaction. Mol Biotech.

[CR24] Min H, Hu SB, Li ZN, Wu YF, Zhang CP, Wei T (2009). A phytoplasma associated with an outbreak of an unusual disease of Chrysanthemum in China in 2008. Plant Dis.

[CR25] Naito T, Tanaka M, Taba S, Toyosato T, Oshiro A, Takaesu K, Hokama K, Usugi T, Kawano S (2007). Occurrence of chrysanthemum virescence caused by “*Candidatus* Phytoplasma aurantifolia” in Okinawa. J Gen Plant Pathol.

[CR26] Pasquini G, Ferretti L, Barba M (2007). Diffusione del legnonerodellavitenel Lazio e caratterizzazione molecolare dell’ agenteeziologico. Inf Fitopatol.

[CR27] Raj SK, Khan MS, Kumar S (2007). Molecular identification of *Candidatus* Phytoplasma asteris associated with little leaf disease of *Chrysanthemum morifolium*. Australas Plant Dis Notes.

[CR28] Raj SK, Snehi SK, Kumar S, Banerji BK, Dwivedi AK, Roy RK, Goel AK (2009). First report of ‘*Candidatus* phytoplasma asteris’ (16SrI group) associated with colour-breaking and malformation of floral spikes of gladiolus in India. Plant Pathol.

[CR29] Rani A, Misra P, Singh J, Kumar P, Rani R, Shukla (2014). PCR based detection of phytoplasma association in pot marigold (*Calendula officinalis* L.) and guldawari (*Dendranthema grandiflora* L.). Asian J Biol Sci.

[CR30] Rao GP, Tiwari AK, Kumar S, Baranwal VK (2014). Identification of sugarcane grassy shoot-associated phytoplasma and one of its putative vectors in India. Phytoparasitica.

[CR31] Rao GP, Madhupriya TV, Manimekalai R, Tiwari AK, Yadav A (2017). A century progress of research on phytoplasma diseases in India. Phytopathogenic Mollicutes.

[CR32] Safarova D, Valova P, Flidr P, Navratil M (2011). Molecular identification of 16SrIII and 16SrXII phytoplasma groups in *Chenopodium album* in Czech Republic. Bull Insectol.

[CR33] Saracco P, Bosco D, Veratti F, Marzachì C (2005). Quantification over time of chrysanthemum yellows phytoplasma (16Sr-I) in leaves and roots of the host plant *Chrysanthemum carinatum* (Schousboe) following inoculation with its insect vector. Physiol Mol Plant Pathol.

[CR34] Schneider B, Seemüller E, Smart CD, Kirkpatrick BC, Razin S, Tully JG (1995). Phylogenetic classification of plant pathogenic mycoplasma-like organisms or phytoplasmas. Molecular and diagnostic procedures in mycoplasmology.

[CR35] Shiomi T, Sugiura M (1983). Water dropwort yellows and Chrysanthemum witches’ broom occurred in Ishikawa Prefecture. Jpn J Phytopathol.

[CR36] Thompson JD, Higgins DG, Gibson TJ (1994). ClustalW- improving the sensitivity of progressive multiple sequence alignment through sequence weighting, position-specific gap penalties and weight matrix choice. Nucleic Acids Res.

[CR37] Tolu G, Botti S, Garau R, Prota VA, Sechi A, Prota U, Bertaccini A (2006). Identification of a 16SrII-E Phytoplasma in *Calendula arvensis*, *Solanumnigrum*, and *Chenopodium* spp. Plant Dis.

[CR38] un Nabi S, Dubey DK, Rao GP, Baranwal VK, Sharma P (2015). Molecular characterization of ‘*Candidatus* Phytoplasma asteris’ subgroup IB associated with sesame phyllody disease and identification of its natural vector and weed reservoir in India. Australas Plant Pathol.

[CR39] Viraktamath CA, Meshram NM (2019). Leafhopper tribe Coelidiini (Hemiptera: Cicadellidae: Coelidiinae) of the Indian subcontinent. Zootaxa.

[CR40] Yadav V, Mahadevakumar S, Sreenivasa MY, Janardhana GR (2015). First report on the occurrence of virescence of chrysanthemum associated with 16SrII-A group phytoplasma in India. Plant Dis.

[CR41] Yadav A, Thorat V, Shouche Y (2016). *Candidatus* Phytoplasma aurantifolia (16SrII Group) Associated with Witches’ Broom Disease of Bamboo (*Dendrocalamus strictus*) in India. Plant Dis.

